# Language effects on bargaining

**DOI:** 10.1371/journal.pone.0229501

**Published:** 2020-03-02

**Authors:** Michael J. Weir, Catherine M. Ashcraft, Natallia Leuchanka Diessner, Bridie McGreavy, Emily Vogler, Todd Guilfoos

**Affiliations:** 1 Dept. of Environmental and Natural Resource Economics, University of Rhode Island, Kingston, RI, United States of America; 2 Natural Resources and the Environment, University of New Hampshire, Durham, NH, United States of America; 3 Department of Communication and Journalism, University of Maine, Orono, ME, United States of America; 4 Rhode Island School of Design, Providence, RI, United States of America; Hanken School of Economics, FINLAND

## Abstract

Language is critical to coordination in groups. Though, how language affects coordination in groups is not well understood. We prime distributive and integrative language in a bargaining experiment to better understand the links between group outcomes and communication. We accomplish this by priming interests or positions language in randomized groups. We find that priming positions as opposed to interests language leads to agreements where controllers, subjects with unilateral authority over the group outcome, receive a larger share of the benefits but where the total benefits to the group are unaffected. In contrast to common justifications for the use of integrative language in bargaining, our experimental approach revealed no significant differences between priming interests and positions language in regards to increasing joint outcomes for the groups. Across treatments, we find subjects that use gain frames and make reference to visuals aids during bargaining experience larger gains for the group, while loss frames and pro-self language experience larger gains for the individual through side payments. This finding suggests a bargainer’s dilemma: whether to employ language that claims a larger share of group’s assets or employ language to increase joint gains.

## Introduction

Language is a defining feature of human interaction. As researchers in the discipline of communication have argued, language as the coordinated use of symbols is, arguably, what makes us human [1]. Communication, through language, plays a vital role in the development of complex rules, social norms, and understandings between and within groups [2–5]. Bargaining, in particular, relies on communication to coordinate group outcomes and organize collective action to solve complex problems, such as climate change or common resource management [6–8]. In addition, bargaining impasses are often a result of information asymmetries [9, 10] and language is used to navigate those issues of fairness and unknown private values. Thus, how language is used to coordinate group outcomes is critical to group and individual performance, evolutionary selection of strategies, and the structure of institutions.

The language used to frame bargaining is essential to how players interpret the interaction and can affect economic outcomes [8, 11–13]. Communication, through specific types of frames, can also be viewed as one of the strategies that players may use to achieve their goals. Experimental research in economics has sought to describe the strategies and outcomes of communication, rather than focusing on the content of the communication [14]. Cheap talk has become a popular way to describe the open-ended and dialogue-based communication in pre-play treatments in these experiments. It is well established that dialogic communication, a.k.a. cheap talk, can overcome barriers (i.e. information asymmetries and uncertainty) to group agreements, leading to greater pro-social behavior and in some cases, more equitable distributions of gains [14, 15]. Communication may also create greater empathy within groups [16], which enhances the ability of groups to overcome a bargaining impasse. Language can be characterized and understood through popular bargaining strategies that parties use in communication.

Distributive and integrative strategies are juxtaposed as critical strategies of bargaining [17, 18], though the literature also uses distributive or integrative approaches to define types of bargaining environments [19]. Distributive and integrative strategies are thought to destroy or create value in negotiations, respectively [17, 20]. Negotiators using distributive strategies, also referred to in the literature as competitive or hard bargaining, focus on achieving their positions and claiming value, but may fail to identify trade-offs that could create more value. Negotiators using integrative strategies focus on both creating joint gains and claiming value to meet their interests relative to negotiators relying on distributive strategies [17, 21]. An integrative negotiation approach therefore requires sharing information about interests and priorities associated with the negotiation at hand. Integrative strategies can then lead to parties identifying and executing trade-offs via negotiation offers to create higher joint gains. These scenarios can result in “win-win” outcomes by combining multiple parties’ interests into a mutually beneficial solution so that all sides claim more value relative to their alternatives, even though some negotiators may claim more relative value than others [22, 23]. Parties primarily use language to convey their messages and arguments to their counterparts to enact their strategies. There is thus a need to understand how communication, and especially the content in dialogue-based interactions, affects negotiation processes and outcomes [24–26].

To unravel the question of how communication influences outcomes through bargaining, we develop a bargaining experiment that tests the priming of distributive and integrative language. Specifically, we randomize groups that play a Coasian bargaining game into treatments that use the language of *interests* or *positions* to prime integrative or distributive language. Coasian bargaining games, where self-interested parties can achieve full efficiency, can have bargaining impasses due to information asymmetries and divergent beliefs about fairness [10, 27]. Therefore, when information is private, it is of particular interest how communication is used to facilitate bargaining. We inspect the outcomes, content of communication, side payments, and efficiency of the bargaining solution between these two treatments. Negotiation facilitators often encourage stakeholders to use integrative language as a way to help a group share information, avoid conflict, and work through disagreements to promote the greatest value to all parties. Despite the prevalence of facilitating integrative language in bargaining, we are unaware of any formal causal tests of the assertion that priming integrative language results in greater joint gains for the group. We find that priming positions does affect communication and the distribution of welfare in favor of subjects with bargaining power, which in our experiment is a single subject’s ability to end negotiations and make decisions unilaterally about the final group outcome (discussed more in “Experimental Design”). The positions prime leads subjects to use more pro-self and loss frames, which in turn increases their share of the rewards being bargained over. We also find that priming integrative language does not increase joint gains in bargaining compared to priming distributive language.

Upon inspection of the content of communication across treatments, we find that subjects who use a gain frame or use visual aids provided in the experiment increase joint gains in their group. This correlative evidence suggests the bargainer’s dilemma: to use loss and pro-self frames which increases your share of a potentially smaller than optimal pie, or to use gain frames which increases joint gains but not necessarily individual returns from bargaining. This concept is also called the negotiator’s dilemma [28] and is more broadly attributed to a negotiator’s decision to employ cooperative or claiming strategies.

Our experiment focuses on the language of bargaining in two distinct ways. First, we use the language of *interests* or *positions* to prime integrative or distributive language in groups. Other experiments that investigate the effect of integrative strategies used experimental manipulations which are broader in scope. These previous experimental treatments affect goal setting by groups which may suggest that subjects are playing either a zero-sum game or an integrative game [29–31]. In contrast, our experimental priming avoids defining the goals of the negotiation but rather allows goals to emerge endogenously from subject preferences and communication. Our study is also different than many other priming studies because we look at language as a mechanism to economic outcomes, rather than priming a social norm or particular identity. Second, we analyze the content of communication to understand further how the use of language leads to different bargaining outcomes. In recognition of concerns about the replicability of priming studies [32] (discussed in more detail in the Methods section), we provide a full description of our experimental procedure and materials to facilitate direct replication of our study as it is, to our knowledge, the only formal causal test of the assertion that priming integrative language results in greater joint gains. Our experimental elicitation allows us to better answer the question: how does communication lead to the creation of bargaining value to groups and individuals?

## Experimental design

In our experiment, three players negotiate over the chance to win a $20.00 cash prize. The chance to win is represented by the number of lottery tickets earned at the end of the negotiation. Since subjects negotiate over the lottery tickets and not the actual cash prize, we are able to control for heterogeneous utilities and risk preferences [33]. This setup also allows utility to be exactly equal to the probability of winning the final cash prize [33, 34]. We use a context specific experiment to allow the use of language to be richer than many other non-contextual economic experiments. The context is chosen to align with a larger project on group decision making processes involving dams, fish passage, and various dam removal options. This scenario is based on a potential dam removal site with the experimental roles representing actual stakeholders in the decision process. Subjects play the role of a Fish and Wildlife representative, a Dam Owner, and a Local Resident. The Dam Owner, the controller, is assigned final authority over the dam scenario decision, giving them rights to end negotiations and unilaterally choose the outcome if no agreement is reached in the negotiation, i.e., exercise “controller’s right”. The Dam Owner’s role as controller allows our lab experiment to more closely mimic the real-world context of a dam removal scenario. The other two stakeholder groups were chosen as roles for this lab experiment as each plays a large role in collaborative bargaining over real-life dam decisions.

As discussed above, stakeholders are often encouraged by facilitators to use integrative language as a way to improve group information sharing, avoid conflict, and work through disagreements to promote the greatest value to all parties. This is particularly true in negotiations over environmental disputes [35]. Recently, stakeholders in New England are involved in environmental disputes related to roughly 14,000 aging dams in the region [36]. As many of these dams approach or exceed their design life, and as preferences for dams and watershed ecosystem services change, society will need to make thousands of decisions about the future of dams in the coming decades. As such, we designed our experiment within the context of a stakeholder negotiation over the potential removal or repair of a deficient dam.

Our study design most closely relates to the economics literature investigating the effect of face-to-face communication on cooperation [37, 38], though the issue faced by bargainers in our experiment is primarily one of coordination and not a social dilemma. Our experiment uses a three-person Coasian bargaining game. The economics literature on bargaining is vast and helps us understand the institutions of bargaining, primarily through information, rules or threats, costs of bargaining, and cheap talk communication [14, 15, 39–42]. We know from this literature that communication generally improves cooperation and that face-to-face communication is a strong and positive moderator of this effect relative to written messages [38]. Our contribution is to understand the mechanics of communication in bargaining more thoroughly through priming language and analyzing content. Most economic experiments have not used priming language coupled with content analysis in bargaining, though facilitating language and using certain frames in negotiations is common practice outside the lab. Our priming treatment is designed to test the causal effect of specific types of language on economic outcomes. We test the following hypothesis:

*H*_1_: *Priming the use of integrative language improves economic efficiency relative to priming the use of distributive language*.

Our expectation that priming integrative language will increase economic efficiency is based on the negotiation literature. There is a large body of literature in the field of communications/negotiation identifying social welfare gains achieved by focusing discussion on partys’ interests rather than positions during a negotiation [8, 11–13, 17, 21]. The economics literature, however, does not predict a difference in outcomes based on integrative or distributive language; pre-play communication is considered “cheap talk” in that the communication has no direct influence on outcome [14]. In short, the two disciplines have differing views on the effects of language in a bargaining context.

The negotiator’s dilemma in our experiment is to maximize individual gains through two primary strategies. First, to signal group outcomes that are beneficial to themselves. Second, to generate better side payment agreements for themselves. These two elements are made jointly. Interestingly, the theoretical solution, presented in the SI, suggests there is no dilemma because socially optimal solutions coincide with the private solutions for individuals. Our analysis suggests that when an individual focuses on maximizing side payments instead of coordinating outcomes, they may leave potential gains on the table by reducing the size of the pie available to the group. This in essence is a failure to coordinate in the group.

### Experimental design details

We recruited 162 undergraduate and graduate students at the University of Rhode Island and University of New Hampshire for participation in the experiment. The sample size was determined based on a power analyses and available resources. We initially calculated the sample size of 30 groups per treatment, which should identify a medium to large sized effect on efficiency with a Cohen’s D of 0.70 with a power of 0.80 and Type I error at 0.05. After running the experiment on 27 groups and upon analysis of the data we collected, it appeared that adding more groups to the sample would not make any appreciable difference to the main hypothesis given the extremely small difference in means between control and treatment. Taking into consideration monetary (per observation) and time costs associated with content analysis, subject recruitment, compensation of research assistants and experimental participants, it is quite costly in money and time to conduct an experiment of this type. This limited the scale of our study. We conducted a total of 27 experimental sessions with a maximum of three participating groups in each. Our lab holds up to three groups simultaneously for this type of experiment. In the event there were three groups in a session, one group negotiated in a small meeting room while the other two groups were placed on opposite sides of our main lab. The main lab is sufficiently large that the two groups could not interfere with one another. Each subject was entitled to a $10 show up fee with the chance of earning an additional $20 if they were the winner of the group bargaining for a total reward of $30 for participation in the sessions which lasted 45-60 minutes on average. Group assignment was randomly assigned. This was done by using a deck of pre-made role assignment cards, which were shuffled and distributed to subjects. Each card told subjects 1) which group they were a part of and 2) which role within their group they would play (Dam Owner, Fish and Wildlife, or Local Resident). Subjects were told explicitly to keep the information on the role assignment cards private. After role/group assignment, subjects completed a short survey to gather basic demographic information and a measure of social motives using the Dutch Test for Conflict Handling (DUTCH) [43]. Next, subjects completed a procedures quiz, followed by a mock transfer of lottery tickets among the three players, and determination of the winner of the final cash prize. The mock transfer of lottery tickets and winner determination provided subject training and experience with the game prior to the experimental session. Following training, the experimenter read aloud a scenario description that outlined a typical setting in which a dam is identified as high hazard and thus prompting stakeholder negotiations. Summary statistics for the sample are provided in Table 1. We conducted t-tests of the difference in means of the observable controls (“Demographics;” “DUTCH Measures;” “Environmental Preferences”) between treatment groups as a check that our randomization was successful. We conclude that, on average, there is no significant difference (*p* ≤ 0.05) between treatment groups among these controls, except in the number of previous experiments. No t-tests were conducted for the C̈ontent” variables as these measures are not based on the randomization.

**Table 1:** DUTCH measures range on a scale from one to five and measure individual conflict handling tendencies. NEP-R score can range from one to five. Scores closer to five imply a more pro-environmental attitude. Content refers to the cumulative number of statements made by a subject during communication sessions in the experiment and coded to the listed categories. A randomization check was conducted for the observable controls (“Demographics;” “DUTCH Measures;” “Environmental Preferences”). Bold variable names indicate a statistically significant difference between treatment groups (*p* ≤ 0.05). We conclude that, on average, there is no statistical difference between our treatment groups, except in the number of previous experiments. No t-tests were conducted for the “Content” variables as these measures are not based on the randomization.

	Position Treatment		Interest Treatment		Full Sample	
	mean	sd	mean	sd	mean	sd
*Demographics*						
Age	22.20	4.69	21.81	4.62	22.01	4.65
Gender (= 1 if female)	0.54	0.50	0.53	0.57	0.54	0.54
**# of Previous Experiments**	1.10	1.53	0.64	1.06	0.87	1.33
*DUTCH measures*						
Yielding	3.04	0.40	3.07	0.40	3.06	0.40
Compromising	3.54	0.69	3.68	0.65	3.61	0.67
Forcing	3.13	0.66	3.12	0.68	3.12	0.67
Problem Solving	3.93	0.65	3.94	0.58	3.93	0.62
Pro-self Tendencies (= 1 if Forcing *Yielding*)	0.43	0.50	0.48	0.50	0.46	0.50
*Environmental preferences*						
NEP-R	3.48	0.31	3.39	0.31	3.44	0.31
*Content*						
Consensus	0.53	0.67	0.36	0.73	0.44	0.70
Use of Visuals	0.65	0.78	0.44	0.88	0.55	0.83
Interest	1.53	1.37	2.06	1.74	1.80	1.58
Position	2.62	1.57	2.02	1.48	2.32	1.55
Pro-self	0.38	0.75	0.19	0.50	0.28	0.64
Pro-social	0.68	0.95	0.44	0.69	0.56	0.83
Gain frame	0.25	0.49	0.25	0.80	0.25	0.66
Loss frame	0.33	0.82	0.14	0.38	0.23	0.65
*Average points earned by role*						
Dam Owner	45.85	15.82	44.70	12.91	45.28	14.31
Local Resident	20.63	11.75	19.30	9.39	19.96	10.56
Fish and Wildlife	22.59	17.02	24.33	14.36	23.46	15.62
*N*	81		81		162	

#### Experimental procedures

**Fig 1:**
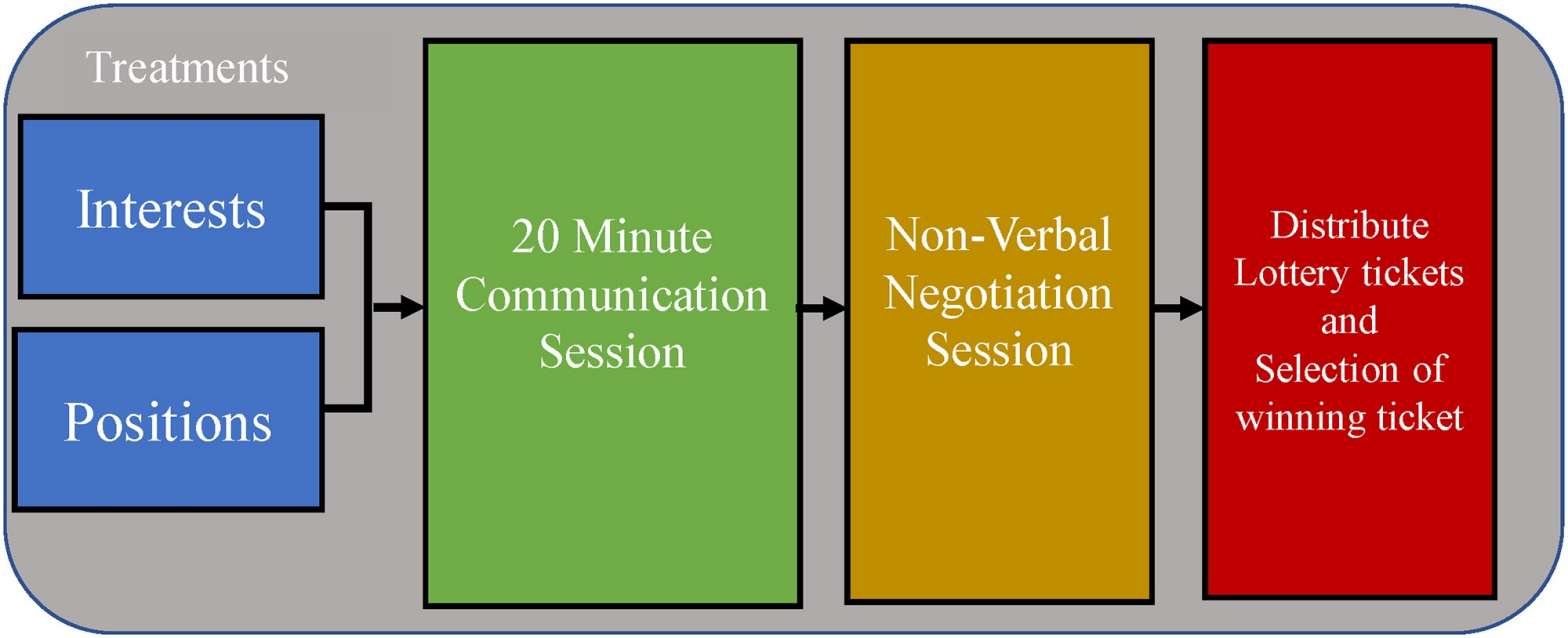
This figure describes the main stages of the experiment. We randomly assign groups to either interests or position treatments. Groups are given instructions and then provided with a 20 minute cheap talk communication session. Then, groups engage in a binding non-verbal offer and acceptance session in which a group decision is unanimously signed onto, or the controller picks the outcome. Last, the points allocated during negotiations are turned into lottery tickets and the lottery drawing occurs and subjects are paid accordingly.

The bargaining game proceeded in three stages, see Fig 1. First, subjects had the opportunity to talk face-to-face among their groups for up to 20 minutes; the groups were not required to use the whole period. after they were provided with the experimental treatment. The experimental treatment was provided in the form of verbal prompts accompanied by physical prime cards with treatment specific primes printed on them, shown in Table 2. Treatment assignment was randomized by experimental session. Randomization by session rather by group within session may increase the noise in the data, because naturally occurring variation in experimental settings can impact outcomes, such as the time of day of an experiment [44]. The experimenter placed the prime cards in front of subjects that represented specific positions or interests followed by the reading of the prompt. The prompt read as follows in the position(interest)-prompted groups, “Pick up 2-3 cards that best reflect the specific options (why you prefer a specific option) for the future of the dam site. Start by discussing your most preferred card choice with the group. As a group, discuss the options for the dam site.” These phrasing differences, though subtle, are intended to suggest to subjects to focus on either what they want (a position) or why they want (an interest) an option given their respective treatment. Aside from the initial prompts, there were no restrictions on subject communication. At this stage, subjects were also directed to visual aids to improve familiarity with the dam site options. Each visual aid showed how the dam site would appear given implementation of each dam site option. These visual aids and experimental instructions are provided in S1 File. The audio from each communication session was recorded and observed by the experimenters. Any agreements made among subjects during the communication session are non-binding.

**Table 2:** List of interests vs. positions were provided to subjects who were prompted to use the terms in negotiations during communication sessions. Each term was printed on a card to help facilitate the use of these frames.

Experimental Treatment Prompts	
Distributive (Positions)	Integrative (Interests)
Repair Dam	Increase Fish Passage
Denil Fish Ladder	Preserve Views of Dam
Nature-like Fish Ladder	Preserve Potential for Hydropower
By-Pass Channel	Protect Property Values
Full Dam Removal	Maintain Cultural Landmark
	Maintain Recreating on Reservoir
	Restore Free Flowing River
	Reduce Risk of Dam Failure
	Make River Accessible to Boats

In the second stage, groups were given 10-minutes to negotiate non-verbally through a mediator and slips of paper which allow for a binding agreement on which dam option is chosen and any side payments between subjects. While participants were still face-to-face during this stage, no verbal communication was allowed during this period. Subjects took turns submitting offers using a provided offer sheet while the experimenter monitored and answered any questions. There was no limit on the number of bargaining offers during this stage. If the offer was determined valid by the experimenter, the offer was displayed for consideration by the other group members. Agreements required unanimous approval in the group. If an agreement was made for a dam option and any transfer of lottery tickets, subjects filled out and initialed a final agreement sheet. This agreement was binding, representing each subject’s final amount of lottery tickets. If time expired, the Dam Owner (controller) was required to exercise their controller’s right. Subjects were then asked to complete a short follow-up survey asking them to reflect on their own bargaining strategies as well as level of environmental concern using the revised New Environmental Paradigm scale (NEP-R) [45]. For clarity, we provide an example bargaining offer and transfer of tickets among subjects.

Subjects negotiated over the dam options listed under Positions in Table 2. Each experimental subject receives a different number of lottery tickets depending on the dam scenario selected and any transfers. Negotiators can receive the default amount of tickets for the scenario, shown in Table 3, fewer tickets if they agreed to transfer some of the points they would have received to another negotiator, or more points if another negotiator agreed to transfer some of the points they would have received. Players may propose a contract to transfer some amount of lottery tickets among the other subjects, which in turn alters the default chance players have to win the final fixed $20.00 cash prize. For example, suppose the Dam Owner proposes the “By-pass Channel” option. Based on the role-specific payoffs and no transfers of lottery tickets, the Dam Owner would get 40 lottery tickets, the Local Resident would get 20 lottery tickets, and Fish and Wildlife would get 15 lottery tickets where the probability of winning the final cash prize is 0.40, 0.20, and 0.15 respectively for each player. Now, suppose there is a transfer of lottery tickets to the Dam Owner of 5 lottery tickets from the Local Resident and 10 lottery tickets from Fish and Wildlife. After the agreed upon transfer, the final probability of winning the final cash prize is now 0.55 for the Dam Owner, 0.15 for the Local Resident, and 0.05 for Fish and Wildlife. Any lottery tickets not allocated among the three subjects are allocated to the “house”. Information asymmetry is imposed by only providing information about one’s own payoffs. No subject was provided with payoff schedules for the other two subjects or the group’s aggregate payoff. The probability of winning the cash prize was utilized during the final stage of the experiment.

**Table 3:** List of the experimental payouts for each group option by subject assigned role. The numbers reported here directly transform into probabilities of winning the lottery cash prize at the end of the experiment. Subjects were provided with information only about their own payouts.

	Payouts			
	Dam Owner	Local Resident	Wildlife Representative	Total
Repair Dam	65	5	0	70
Repair & Denil Fish Ladder	55	5	15	75
Nature-like Fish Ladder	35	25	40	100
By-Pass Channel	40	25	20	85
Full Dam Removal	0	10	70	80

In the third stage, once the post-experiment surveys were complete, the experimenter randomly drew one lottery ball using a bingo cage to determine the winner of the final $20 cash prize. Once the winner was determined, subjects received cash payments as they left the lab. The number of subjects that won the cash prize could vary from zero to three depending on the group option selected and any ticket transfers agreed upon.

## Methods

The University of Rhode Island Institutional Review Board approved this project, HU1516-003. Written consent was obtained from subjects.

### Priming studies

Our study relies on a prime to test our experimental hypothesis. While this is not a new technique, the effectiveness of priming as a treatment mechanism and the replicability of reported priming effects has been critiqued in the behavioral sciences in recent years. We discuss each of these considerations in turn.

In our study, one concern is that our experimental subjects did not interpret our primes as we designed them. To address this concern, we recruited two independent research assistants to gauge the interpretation of our priming treatment by the experimental subjects. Specifically, we recruited the analysts to review our fourteen priming statements (five position statements, nine interest statements) and categorize them as either “interests” or “positions.” We compared their responses to our priming designations presented in the manuscript. The first analyst coded all fourteen statements in agreement with our designations, while the second analyst correctly coded thirteen of the statements in agreement with our designations. Overall there is 95.2% agreement among the coders and our designation with Krippendorf’s alpha equal to 0.901 which is indicative of strong internal consistency. The results of this analysis give us confidence that any priming effects observed in fact address the proposed hypotheses.

A related, though separate, methodological concern is the replicability of priming studies. Since the early work by Bargh, Chen and Burrows [46] reporting strong behavioral effects of subtle primes, the prime-to-behavior literature has grown substantially [47]. The body of work dedicated to replicating reported priming effects, however, is much smaller and comprised of primarily *conceptual* rather than *direct* replications [48]. Those replications that are available attempt to replicate effects based on studies with low statistical power related to small sample sizes. As such, the minimal number of (direct) replications of priming effects results in a lack of “well-developed formal theories of priming that can pinpoint exactly what features and what effects should be important” [32]. In other words, researchers cannot fully distinguish between the necessary and sufficient features of a priming study for replication attempts. Cesario [32] and Kahneman (in his open letter) both propose more direct replication by multiple independent labs is necessary to understand generalizability of priming effects and diligence by priming researchers to exercise conservatism in reporting effect sizes.

In recognition of these criticisms, we provide a full description of our experimental procedure and materials to facilitate direct replication of our study as it is, to our knowledge, the only formal causal test of the assertion that priming integrative language results in greater joint gains. We also note that our test of the primary hypothesis produces a null result. We did not publish a pre-analysis plan for the work herein and recognize the issues of not doing so, particularly those associated with p-hacking and data-mining. Thus, we are fully transparent about components typically included in a pre-analysis plan throughout this manuscript: outcome variables, variable definitions, inclusion rules, model specification, co-variates, and subgroup analyses [49].

We also conduct post-hoc power analyses [50]. We refer to Andersson et al.’s [51] reported effect size estimates to inform our analysis as it is, to our knowledge, the most closely related work. However, the study is only loosely related to the work herein as it is not a bargaining study and the research design utilizes a different priming mechanism. The standardized effect sizes reported in Andersson et al. [51] are 1.36 in their full sample analysis and 2.96 in their split-sample analysis. We estimate statistical power for these effect sizes (0.26, 0.83) assuming a standard error = 1 and degrees of freedom = 52. For both effect sizes we have and a 1% chance of our significant effects being the wrong sign. The “type-M” exaggeration factor is 1.92 for the full sample and 1.10 for the split-sample analysis.

### Econometric methodology

We investigate our results using mixed methods. We use content analysis to investigate the content of communication during bargaining and regression analysis to understand the impact of treatment and content on bargaining outcomes. The primary results are presented in terms of treatment effects by simple comparison of means. We further investigate the differences in means using additional controls and the ordinary least squares (OLS) model. The two outcomes of interest are economic efficiency and the size of side payments made between subjects.

We measure the outcome of bargaining in terms of reward efficiency which represents the improvement in actual expected gains as a percentage of the potential gain from bargaining as defined in Eq 1.

$$\begin{array}{c} R=\frac{{\hat{p}}_{A}+{\hat{p}}_{B}+{\hat{p}}_{C}-p_{A}^{o}}{1-p_{A}^{o}} \end{array}$$ (1)

where ${\hat{p}}_{A}$, ${\hat{p}}_{B}$, and ${\hat{p}}_{C}$ represent a subject’s chance to win the final cash prize given their role. The outside option for the controller is 65 lottery tickets, the maximum number of lottery tickets they can receive if they exercise the controller’s right. Efficiency is maximized when all 100 lottery tickets are distributed amongst the subjects. When subject’s fail to obtain agreements with full efficiency the “house” (experimenters) retains the lottery tickets that are forgone.

To find the effect of treatment or additional controls on economic efficiency we estimate Eq 2 for observation *i*, *X*_*j*,*i*_ and a vector of *J* explanatory variables, and *ϵ*_*i*_ is the error term.

$$\begin{array}{c} R_{i}=\beta_{0}+\sum_{j=1}^{J}\beta_{j}X_{j,i}+\epsilon_{i} \end{array}$$ (2)

A similar equation is estimated to analyze side payments using Eq 3; for observation *i*, *X*_*j*,*i*_ and a vector of *J* explanatory variables, and *ϵ*_*i*_ is the error term.

$$\begin{array}{c} SidePayments_{i}=\beta_{0}+\sum_{j=1}^{J}\beta_{j}X_{j,i}+\epsilon_{i} \end{array}$$ (3)

### Mediation model

We also conduct an exploratory analysis investigating the pathway in which treatment affects economic outcomes through a mediation model following Preacher and Hayes [52]. This is accomplished by estimating the effect of treatment on the content of communication through a variable, *M*. *M* is a *mediator* if treatment affects the economic outcome variable indirectly through *M*. As we are interested in understanding the mechanisms through which language affects bargaining outcomes, this method allows us to isolate the causal pathways of treatment on the economic outcomes of efficiency and side payments. To estimate the direct and indirect effects of our treatment through language content we estimate Eqs 4 and 5.

$$\begin{array}{c} OutcomeVariable_{i}=\beta_{0}+\beta_{1}*Treatment+\beta_{2}*M_{i}+\sum_{j=3}^{J}\beta_{j}X_{j,i}+\epsilon_{i} \end{array}$$ (4)

$$\begin{array}{c} M_{i}=\alpha_{0}+\alpha_{1}*Treatment+\sum_{j=2}^{J}\alpha_{j}X_{j,i}+\xi_{i} \end{array}$$ (5)

In Eqs 4 and 5, *M* is a specific language concept that we code and think has an effect on the outcome variable. The variables represented by *X*_*j*,*i*_ are additional control variables in the regression. The direct effect of treatment on the economic outcome of interest is captured by *β*_1_ in Eq 4. The indirect effect of *M* is the product of *α*_1_ and *β*_2_. *M* is a mediator if this product is statistically different from zero.

### Content analysis

We develop a codebook, available in Table K in S1 File, that we use to code speech between subjects during the communication stage of the experiment to specific themes that address our research questions. This analysis is meant to capture important signifiers in language that may direct or shape a negotiation. Our coding scheme focuses heavily on aspects of argument *framing* as it is considered to play an important role in shaping the evolution of environmental issues [8, 35, 53]. How a situation is framed, by potential disputants and/or observers, can define whether a problem exists and precisely what the problem is [54]. We capture the gain or loss framing as it is an established argument frame used in practice, particularly in analyzing case studies involving environmental disputes [35, 55, 56]. We also capture subjects’ use of data or visuals provided during the experiment, moments of consensus, power frames, social preferences, and most importantly the interests and positions. Interests or positions are identified by matching any of the interest or position primes described above in Table 2 with dialogue of a subject. The number of observations per statement type varies between subjects. As such, it is possible for certain statement types to occur with minimal frequency. We do not present other concepts that are coded if they are insignificant in frequency and do not lend themselves to analysis.

Practically, content analysis requires the analyst to review transcripts for each session and assign a category to subject statements that align with the pre-defined definition. So, not all statements in a transcript are coded within one of the categories. In the event there are multiple consecutive statements closely related to a single content category, the group of statements is coded as a *single* statement. Thus, we assume multiple related statements presented consecutively to be a part of the same argument. This observation is rare, however. In the more likely case of multiple non-consecutive statements closely related, each statement would be coded as separate statements. Taken together, this coding approach results in a downward-biased measure of statement frequency and is thus more conservative.

To help understand the categories of coded communication, we provide the following examples. *Consensus* statements indicate that the group is converging on agreement. For example, “I feel like we’re all in agreement about the bypass channel.” Statements coded as *use of visuals* refer to those in which subjects specifically mention aesthetics of a dam site option or generally refer to the provided visual aids, including the prime cards. For example, “…I would prefer to see the nature-like fish ladder because it looks more like nature and isn’t such an eyesore with that road running across it and these cement walls.” *Interests* or *positions* statements make reference to the interests or positions that the experimenters provided in Table 2. An example of an *interest* statement is, “I think it’s most important to maintain the fish population, get it back to where it should be,” while an example of a *position* statement is, “I would like to have bypass channel.” *Pro-social* statements like, “You’re also allowing more fish to come, which will help the residents who are doing the fishing,” indicate a point of view for the others in the group or for the group itself. *Pro-self* statements like, “I like the bypass channel, as the controller because I still keep the dam,” indicate a focus on the self or self-interest. *Gain* coded statements refer to the potential gains to a subject when discussing options. A statement such as “So I would be getting 40 [points] for the nature-like fish ladder” would be coded as a *gain* frame. Statements referring to losses from implementing a specific dam site option are coded as a *loss* frame. For example, statements like, “The income I would get from hydropower and the land that I would be losing because of taking and removing the dam completely would just be a lot of loss on my part,” are coded to the *loss* frame.

Communication sessions were recorded on digital tape recorders and were transcribed through a professional transcription service. Since subjects were asked to say their role before speaking the transcriber was able to assign roles to the speaker. Coding was executed by the lead author, blind to the treatment during coding, who independently coded each statement to a particular category based on the codebook utilizing NVivo 12, a qualitative analysis software used to store, manage, and analyze large qualitative datasets. Frequency of each code was identified by the number of coding references in NVivo. The coding spread across negotiating groups and the individual roles within each group was derived using a crosstab query in NVivo. Crosstab results were exported into Excel, and then prepared for statistical analysis in STATA.

An independent coder, blind to experimental design, reproduced the coding scheme for 50% of the sessions to assess intercoder reliability. We find a fair level of agreement among categories of coding: 94.2% agreement on consensus, 77.2% agreement on gain frames, 86.7% agreement on loss frames, 94.6% agreement on use of visuals, 81.3% agreement on interests, 83.8% agreement on positions, 96.9% agreement on pro-self, and 91.6% agreement on pro-social statements.

## Results

We assess the effectiveness of the priming treatment by comparing the percentage of interest based statements between treatments (0.485 vs. 0.331), which is significantly different using a two-sample t-test (p = 0.0005) and is robust to inclusion of additional controls and multi-variate regression analysis (demographics and preferences) as shown in S1 File. Further, the distribution of dam options outcomes between treatments is available in Fig A in S1 File.

**Fig 2:**
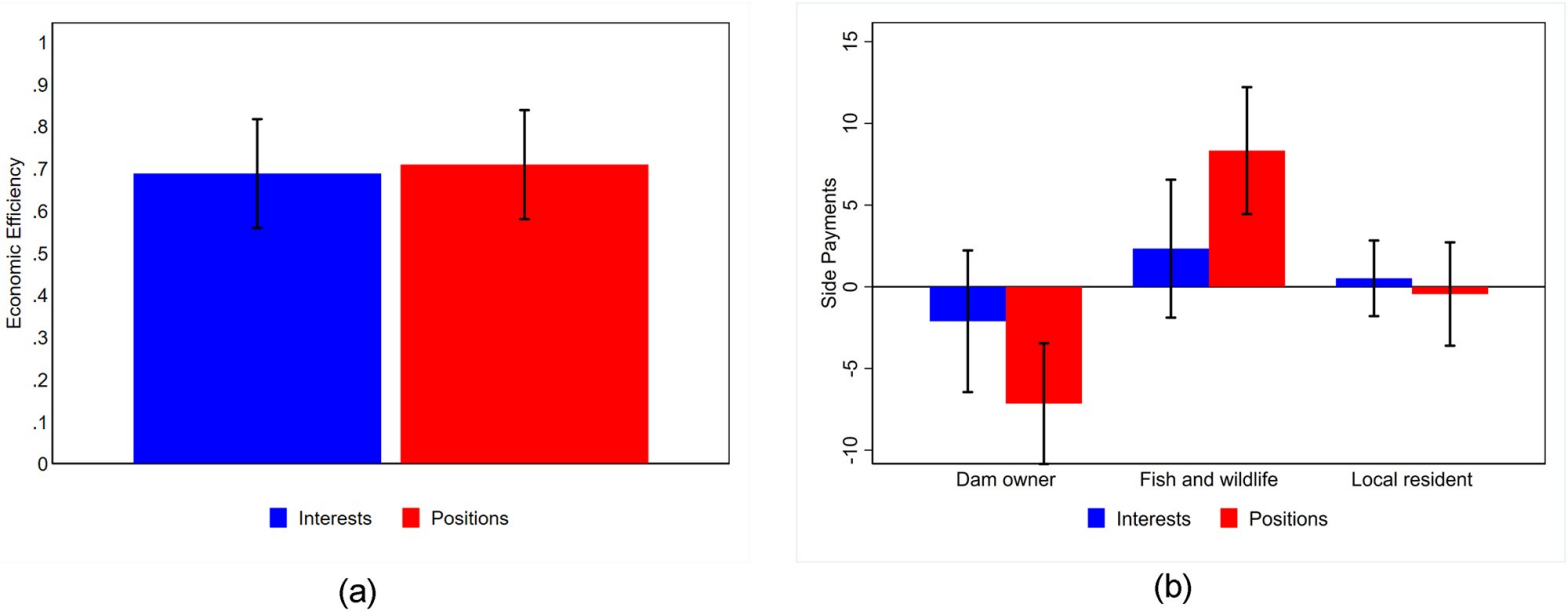
Fig 2a shows economic efficiency of group decisions by treatment. Fig 2b shows the average side payments made by role by treatment. Standard errors are shown at 5% with 27 observations in each treatment in each role.

Fig 2a shows no significant difference between the interests and positions treatments on economic efficiency, a measure of joint gains for the group. The difference in means is statistically and economically insignificant using a two sample t-test (p = 0.8171). Economic efficiency is defined, in the Methods section, by Eq 1, which is the gains available to groups from bargaining. We show in Table A in S1 File that the treatment effect is robust to inclusion of controls (demographics and pro-social preferences) in a multivariate econometric design estimating Eq 2. One potential reason for null results of the experiment could be that the level of statistical power is insufficient to detect a small treatment effect. However, given the extremely small difference in means between our control and treatment groups it is not surprising that these differences are statistically insignificant under various definitions of economic efficiency or controls used in the analysis. Further, there are no differences in the number of early stoppages between treatments. Thus, we are less concerned that statistical power is driving the null result.

These results confirm our prior understanding of distributive language as a claiming focus for bargainers but do not support hypothesis *H*_1_: that integrative language increases joint gains for the group.

Fig 2b demonstrates the effect of priming positions on the distribution of side payments. In this figure, a negative value denotes receiving a side payment from other group members and a positive value denotes giving a side payment to other group members. Dam owners, who have bargaining power derived from a strong alternative (they have the ability to end negotiations and make decisions unilaterally), receive larger side payments in the positions treatment which primarily come from the Fish and Wildlife subjects, presumably because the Fish and Wildlife role has greater ability to pay than the Local Resident role. The difference in the Dam Owner’s side payments by treatment is significant at the 10% level using a two-sample t-test (p = 0.0867) and at the 5% level for the Fish and Wildlife subject (p = 0.0438). A focus on positions directs a greater distributional effect on bargainers which leaves subjects with bargaining power a greater share of the pie. The mechanism for this is likely the increase in loss and pro-self statements from Dam Owners which we show in the communication analysis and in S1 File.

One benefit of our study is that we designed it to capture the differences in communications between subjects. To further understand how the communication used by subjects during bargaining affects bargaining outcomes, we analyze the content of the communication sessions.

### Bargaining language

We analyze how the content of the communication affects two outcomes from bargaining. First, we explore which communication strategies are associated with joint gains for the group. Second, we explore which communication strategies affect the distribution of points through side payments between subjects. We want to emphasize that this analysis is a correlation analysis and is not causal since bargaining outcomes are jointly determined with content of communication. The issue is that omitted variables, such as personality types, could drive both the outcomes and content of communication.

**Fig 3:**
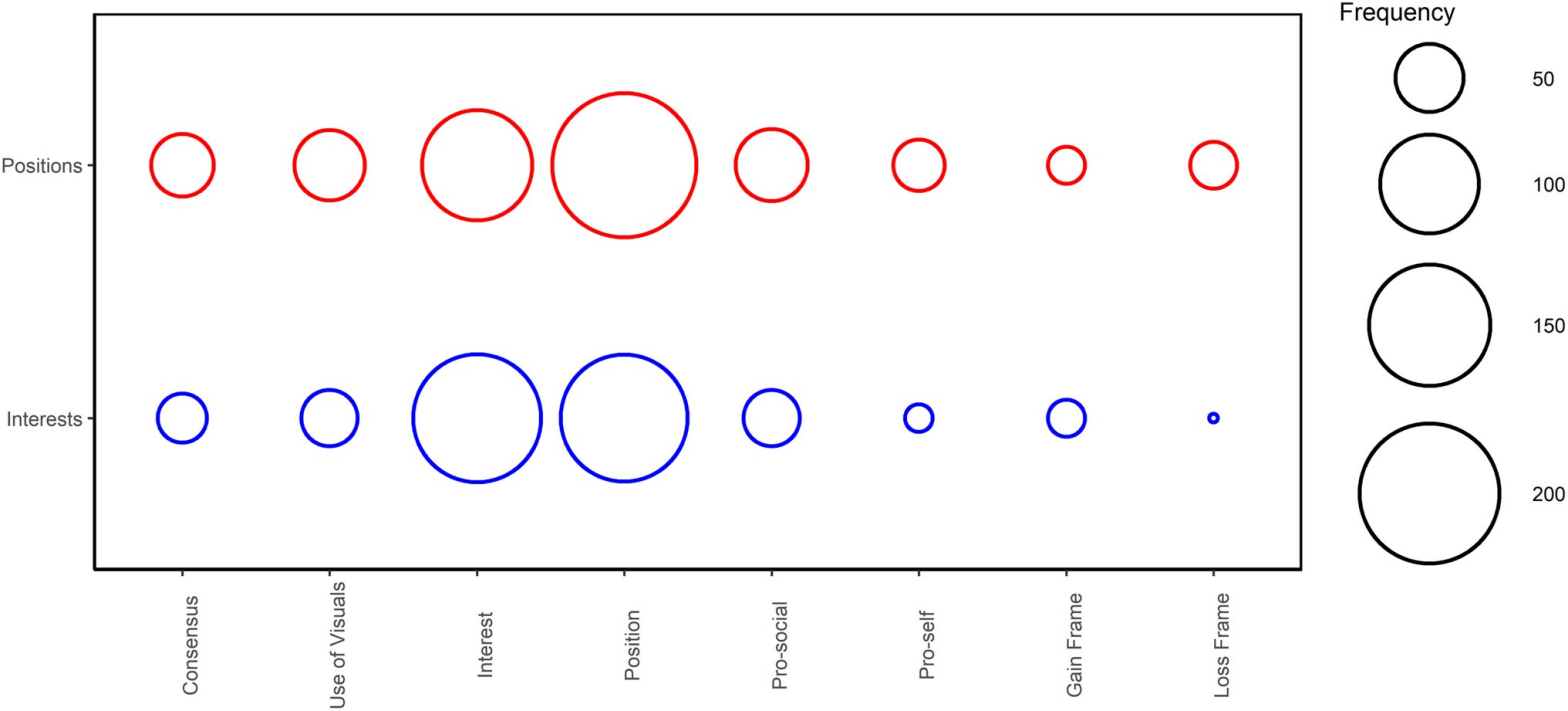
Content of communication. This figure shows the relative frequency of the content of various communication used by subjects during the communication phase of the experiment.

Fig 3 shows the reference frequency of communication concepts coded as described. This figure demonstrates that groups between treatments were fairly similar in their communication, though some differences are noteworthy. As expected, groups primed with *interests* use interest based statements more often. Subjects primed with *positions* were more likely to use a *loss* frame and *pro-self* statements more often than the subjects primed with *interests*. The difference in the mean number of *pro-self* (0.383 vs. 0.185) and *loss* statements (0.333 vs. 0.136) between treatments is just above 5% significance using a two-sample t-test (p = 0.0509) and (p = 0.0512), respectively.

In environmental disputes, like the case we represent in our experimental design, it is common that parties see actions taken by others as resulting in either a personal loss or gain [35]. We suggest that position groups used the *loss* and *pro-self* frame more frequently as a result of our position prime inducing the use of competitive (distributive or value claiming) strategies. In this sense, subjects in the position groups had clear individual reference points for their desired dam site outcomes and were nudged into more claiming strategies. Our findings that loss frames are associated with more competitive behaviors is consistent with findings from other research that negotiators are more risk seeking when decisions are framed as losses and therefore more likely to engage in value claiming [57].

**Fig 4:**
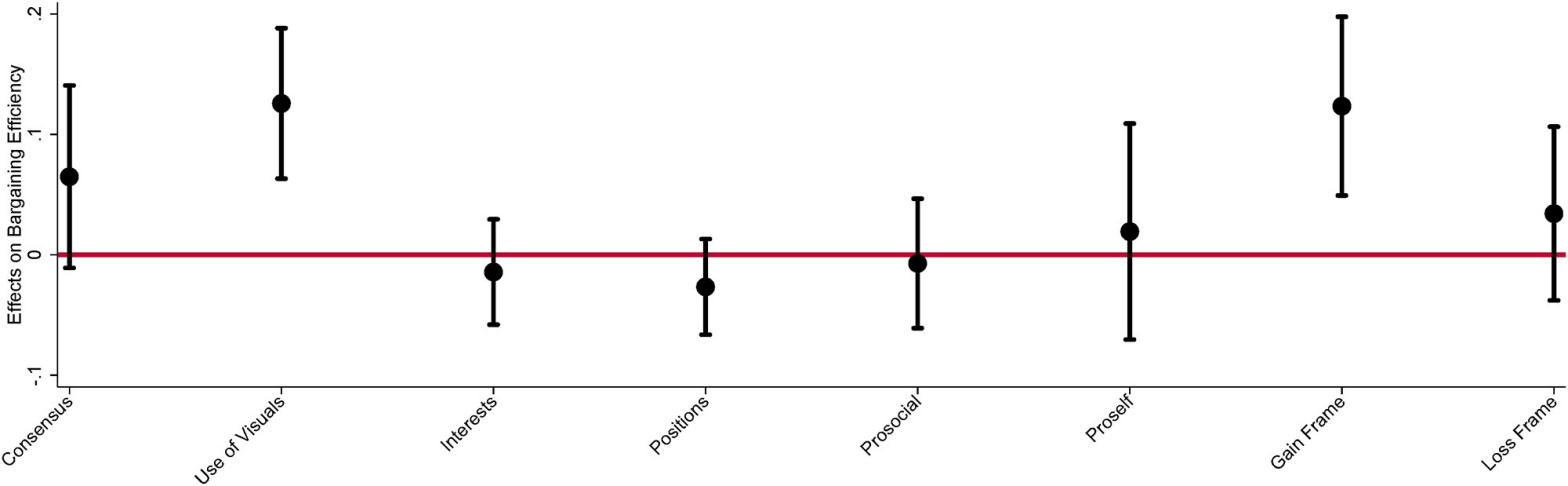
Effect of communication on value creation. The estimates from an OLS model are estimated and the marginal effects of subject’s use of communication is measured against bargaining efficiency, defined in Eq 1. N = 162 and standard errors are clustered at the group level. The confidence intervals on the figure are calculated at 95%. These results are estimated without additional controls, models with additional controls are available in Table J in S1 File.

To estimate the effect of communication content on economic efficiency, as defined in Eq 1, we use an OLS model given in Eq 2. Fig 4 illustrates the marginal effect on bargaining efficiency of an increase in a subject’s use of the communication concept. For this analysis a subject’s statements are coded then aggregated for that subject over the communication session and the frequency of statements is used as an explanatory variable in the OLS model. The marginal effects on efficiency are presented here without additional controls, demographics and preferences. We include results with additional controls in S1 File. Fig 4 shows subjects using a *gain* frame or *use of visuals* in their bargaining language increase the bargaining efficiency of their groups. To a lesser extent subjects that use *consensus* statements gain bargaining efficiency but with a weaker statistical significance.

**Fig 5:**
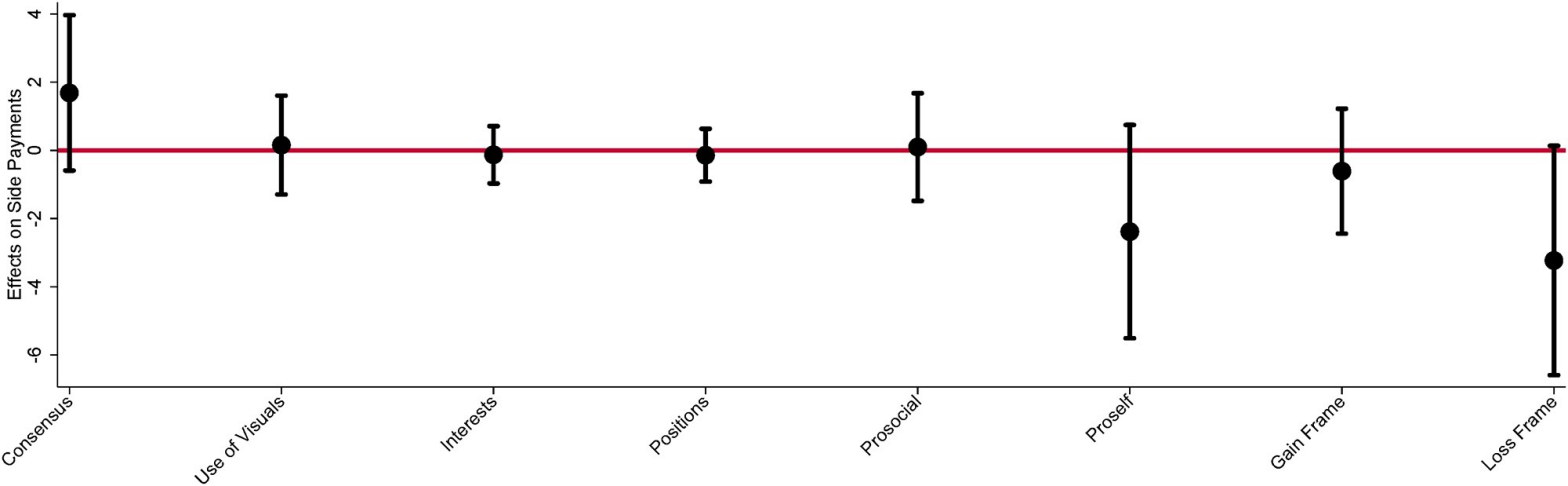
Effect of communication on side payments. The estimates from a linear OLS model are estimated and the marginal effects of subject’s use of communication is measured against the associated side payment of the subject. N = 162 and standard errors are clustered at the group level. The confidence intervals on the figure are calculated at 95%. These results are estimated without additional controls, models with additional controls are available in Table C in S1 File.

In Fig 5, we illustrate how the distribution of gains to a subject are affected by the content of their communication. Subjects that make *pro-self* and *loss* frame statements capture greater side payments from other group members. This aspect of distributional welfare does not increase the gains to their group but rather comes at the expense of other group members. This is further confirmation that loss frames lead to more competitive negotiation strategies. The marginal effects on side payments are presented here without additional controls, demographics and preferences. We include results with additional controls in S1 File. We note that statistical precision of these estimates are weak and sometimes not significant at the 10% level. We take note in them due to the combined difference in concepts between treatments and the association with distributional aspects by role of the subject. To further assess content through the priming treatment we use mediation analysis.

#### Mediation analysis

As described in the Methods section, we conducted a mediation analysis to test the pathways of our treatment effects through content of language. Since we cannot reject the null hypothesis that our treatment has no effect on economic efficiency we do not perform the analysis on economic efficiency. For side payments, we estimate the models defined by Eqs 4 and 5, controlling for *loss frame* and *pro-self* statements to identify if treatment priming is related to an indirect effect on bargaining outcomes through these language frames. We choose *loss frame* and *pro-self* because these concepts are correlated with increases in side payments received as found in Fig 5. In Table 4, we find that *pro-self* statements have a weak relationship to side payments for Dam Owners, which is not significant at the 10% level. We also find some evidence that is consistent with *loss frame* having an indirect effects for Fish and Wildlife subjects. While there is some weak evidence of the pathways where the position treatment is associated with the distribution of welfare through side payments, the lack of power in our design likely leads to imprecise estimates of these associations.

**Table 4:** Mediating role of language on side payments. Standard errors in parentheses. * p < 0.1, ** p < 0.05, *** p < 0.01.

	Dam Owner	Fish and Wildlife	Local Resident
Direct effect of positions treatment	-4.394 (3.322)	4.816 (3.015)	0.562 (2.079)
Indirect effect of position treatment → Loss statements	-0.704 (1.505)	-1.476* (0.842)	-0.335 (0.442)
Indirect effect of position treatment → Pro-self statements	-1.917 (1.529)	-0.010 (0.096)	0.160 (0.281)
Demographic controls	Y	Y	Y
*N*	51	51	52

## Discussion

We find that language is likely important to economic outcomes of subjects in bargaining. Specifically, we find that subjects use of *gain* frames or *use of visuals* is correlated with additional value to their groups. This language may be related to integrative strategies through the pathway of information sharing. Pruitt [23] identified three primary integrative strategies implemented in negotiations: explicit information sharing, implicit information sharing, and heuristic trial and error. Subjects use of the provided visuals during exchange of questions and answers, a form of explicit information sharing, helped our negotiating groups gain insight about other parties’ interests and priorities. This explicit sharing of information resulted in higher bargaining efficiency, i.e., greater joint gains, and is consistent with previous findings [58]. Further, the use of a gain frame may be an aspect that allows greater concessions and increases group outcomes, which is also consistent with previous evidence [59]. Communication that identifies potential gains and uses visual arguments is correlated with success in reaching better economic outcomes for the group.

Priming distributive language in bargaining has important distributional effects. Through exploratory analysis, we observe subjects using more *loss* frames and *pro-self* frames when primed with positions language. Our position treatment is correlated with subjects with bargaining power claiming a greater slice of the bargained for pie at the expense of other group members. We find weak evidence that the position treatment is associated with indirect effects of *loss* and *pro-self* statements which increase side payments for certain subjects. Our results are consistent with other research that finds power fosters value claiming in negotiation [60], that lower power parties are more likely to drive value creation [61], and that competitive context affects bargaining power, as compared to cooperative contexts [62, 63]. Specifically, we observe Dam Owners, parties who have higher power derived from better alternatives, receiving more side payments and therefore claiming more value. Fish and wildlife, a lower power party, was more likely to engage in integrative bargaining and strive to create value by offering more side payments. And, interest and position priming seem to reinforce these effects of unequal power. Dam owners in negotiations receive larger payments in the positions treatment, as compared to the interests treatment. Fish and wildlife offered larger side payments in the positions treatment, as compared to the interest treatment.

We find suggestive evidence for a bargainer’s dilemma from the language used by bargainers, based on the correlation of language used and economic outcomes. In Tables H and I in S1 File we show that there is a small overlap in *pro-self* vs. *gain* frames or *pro-self* vs. *pro-social* communication concepts. Bargainers balance which communication strategy to use to increase gains for themselves. While *pro-self* language is associated with larger individual gains, it is not associated with an increase in the gains for the group. While *gain* frames are associated with an increase in joint gains for the group they may not improve gains for the individual relative to the claiming strategy of pro-self statements. This result is consistent with previous work on positive vs. negative frames in integrative bargaining settings [11] and suggests that framing or language that benefits both the group and individual may not overlap.

In our main hypothesis, we find a lack of evidence to support the causal inference that integrative language creates more value for the group than distributive language through the priming of interests and positions. There are a few possible explanations for this finding. First, perhaps our particular facilitation of bargaining strategies is ineffective, but other integrative interventions are more effective at creating value. Nonetheless, it could still be that our facilitation technique alone is not enough to prime integrative language. Other experiments have found positive effects of manipulations that include exposing subjects to negotiation strategies that go beyond priming integrative language [30, 31], which includes tactics such as goal setting and different integrative frames. There is concern that these types of manipulations (i.e. goal setting) are more effective in the lab than in the field, as subjects may be complying to experimenter demands as to what constitutes acceptable or appropriate behavior [64]. It is difficult to dismiss the idea that integrative language can be effective in conjunction with other negotiation strategies. Second, our experiment is particularly costly to run and analyze through content analysis per observation and therefore lacks comparable power to other economic experiments. Third, integrative strategies are difficult to facilitate. It is worth noting that we did not have anyone play the role of facilitator in this experiment, which could have provided stronger effects by nudging groups continually during the communication sessions. Though, we show evidence, in Fig 3, that we are able to move language used by subjects towards integrative concepts by priming interests. Lastly, it could be that priming integrative language does not create significant economic gains. More substantive designs are necessary to realize economically significant gains from bargaining. While the findings presented here do not support the hypothesis that priming integrative language increases economic efficiency in bargaining outcomes, more work needs to be done to explore variations and the robustness of this finding.

The significance of being able to induce language in bargaining is to establish the causal relationship between aspects of particular bargaining strategies and outcomes. If the strategies themselves are correlated with traits, pro-social preferences, or other structural features of the negotiation, then the aspects of strategies may appear to be causal when they are a function of another underlying cause. Through the random assignment of two induced language manipulations to experimental groups, we formally test the causal link between integrative and distributive language and certain bargaining outcomes. Our experiment is designed to assess the relationship between induced bargaining language in the absence of other essential bargaining tactics. Our finding suggests that care should be taken in interpreting communication from negotiations, and to treat the aspects of integrative bargaining strategies as separate but important processes.

## Supporting information

S1 File Supplementary information for language effects on bargaining. (PDF)
