# Supplementary material for: Language effects on bargaining
Source: PLoS One. 2020 Mar 2;15(3):e0229501. doi: 10.1371/journal.pone.0229501 (PMC7051044; doi:10.1371/journal.pone.0229501)
Supplement: S1 File — (PDF) [file pone.0229501.s001.pdf]

Supplementary Information: Language Effects on Bargaining

February 16, 2020

1 Bargaining outcome summary

Experimental data analyzed in the manuscript and SI is available on figshare: https://doi.org/10.6084/m9.figshare.11591565.v1.

Figure A summarizes the distribution of bargaining outcomes by treatment. The distribution across treatments is similar, giving us some additional confidence in the inference made from our econometric methods.

2 Nash Equilibrium

Here we define Nash Equilibrium for each role. Note that full economic efficiency is reached in the Nash Equilibrium solution. Assume we have three risk-neutral bargainers, Players A, B, and C, where Player A is the controller. Let p_A be the negotiated probability player A wins the large or small cash prize, Z > z = \$ 0. Normalizing utility such that u(Z) = 1 and u(z) = 0, player A's expected utility from a negotiated agreement is $EU_A = p_A u(Z) + (1 - p_A)u(z) = p_A$. Define p^o_A as the probability player A win's the large cash prize given they exercise their controller right. Then, player A's expected utility from exercising their controller right is $EU^c_A = p^o_A u(Z) = p^o_A$.

Let p_B and p_C be the probability player B and player C win the large cash prize, respectively. Then, player B's expected utility from a negotiated agreement is equal to $EU_B = p_B u(Z) + (1 - p_B)u(z) = p_B$, and similarly, player C's expected utility from a negotiated agreement is $EU_C = p_C u(Z) + (1 - p_C)u(z) = p_A$.

Now, let $p_H = 1 - p_A - p_B - p_C$ be the probability no player wins the final cash prize from a negotiated agreement. If the controller exercises

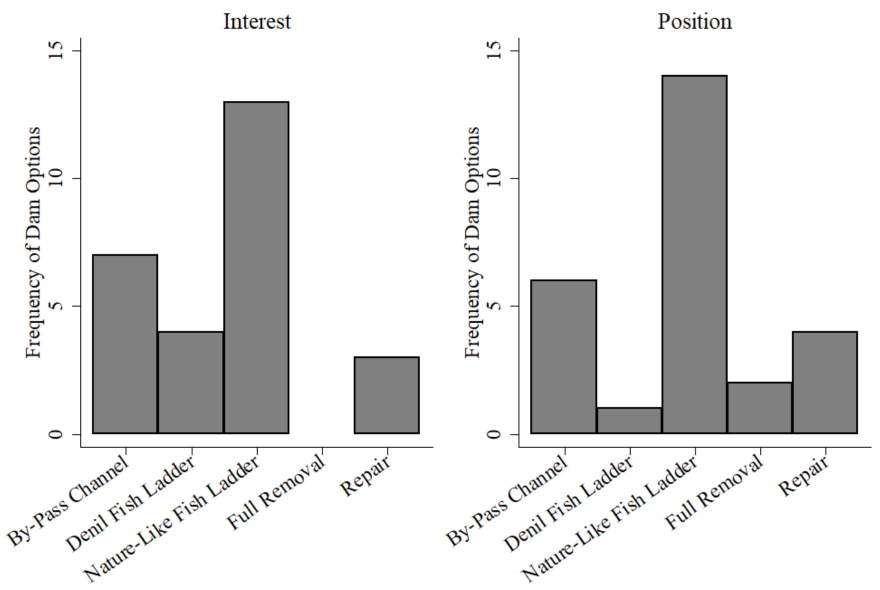

Figure A: Frequency of bargaining outcomes by treatment

their controller right, player B has a non-zero outside option of winning the large cash prize, p_B^o , while player C has an outside option of zero. So, expected utility measures for player B and player C are $EU_B^{nc} = p_B^o u(Z)$ and $EU_C^{nc} = (1 - p_A - p_B - p_H)u(z)$. The Nash bargaining problem for our experiment is given in equation 1.

$$max_{p_A,p_B,p_C,p_H}(EU_A - EU_A^C)(EU_B - EU_B^{nc})(EU_C - EU_C^{nc}) \tag{1}$$

The solutions to the maximization problem above are provided in equations 2, 3, and 4

$$p_A^* = \frac{1 + 2p_A^o - p_H - p_B^o}{3} \tag{2}$$

$$p_B^* = \frac{1 - p_A^o - p_H - 2p_B^o}{3} \tag{3}$$

$$p_C^* = \frac{1 - p_A^o - 2p_H - p_B^o}{3} \tag{4}$$

Given our experimental setup, the outside options for player A and B are $p^o_A = 0.65$ and $p^o_B = 0.05$. The above expressions are maximized when all 100 lottery tickets are distributed among the three players ($p_H = 0$)and thus the Nash equilibrium bargaining predictions are $p^*_A = 0.75, p^*_B = 0.15$, and $p^*_C = 0.10$.

Note, that our game could be analyzed as a Bayesian Nash Equilibrium though we do not know the prior distribution of beliefs to establish solutions for our empirical results. We do not investigate this primarily because deviations from any Nash Solution would not be important to our treatment effect of priming and is not the focus of this research.

3 Analysis of Results

The results from the main text are expanded on in this section to include more explanatory controls. The basic regression uses a an ordinary least squares (OLS) model as in equation 6 where the reward efficiency is defined in equation 5.

$$R = \frac{\hat{p}_A + \hat{p}_B + \hat{p}_C - p^o_A}{1 - p^o_A} \tag{5}$$

$$R_i = \beta_0 + \sum_{j=1}^{J} \beta_j X_{j,i} + \epsilon_i \tag{6}$$

When evaluating group efficiency we estimate equation 6 for observation i, $X_{j,i}$ is a vector of J control variables, and ϵ_i represents the error term.

3.1 Economic Efficiency

To analyze the treatment effect of priming integrative language we run the regression on equation 6 with observations at the group level and include increasing controls in Table A. The treatment effects of position groups versus interest groups is economically and statistically insignificant in all specifications, consistent with our findings in the main text. This finding is robust to controlling for the number of bargaining offers during the non-verbal negotiations, see Table B.

Throughout the rest of the SI we will refer to demographic controls as the age, gender, and previous number of experiments, and preference controls as responses to survey questions that capture preferences, pro-social and pro-self preferences, interpersonal conflict behaviour through the DUTCH survey

[Van der Vliert, 2013], and environmental preferences through NEP-R survey [Dunlap et al., 2000].

	(1)	(2)	(3)
Positions treatment	0.0212	0.0763	0.0212
	(0.0910)	(0.114)	(0.127)
Lab Location FE	N	Y	Y
Preference controls	N	Y	Y
Demographic controls	N	N	Y
N	54	53	53
R^2	0.0010	0.0518	0.1449

* p<0.1, ** p<0.05, *** p<0.01. Standard errors are in parentheses.

Table A: OLS Model: Effect of treatment on economic efficiency

	(1)	(2)	(3)
Positions treatment	0.0289	0.0760	0.0245
	(0.0915)	(0.114)	(0.129)
Number of bargaining offers	-0.0116	-0.0107	-0.00420
	(0.0123)	(0.0138)	(0.0150)
Lab Location FE	N	Y	Y
Preference controls	N	Y	Y
Demographic controls	N	N	Y
N	54	53	53
R^2	-0.0205	-0.157	-0.168

* p<0.1, ** p<0.05, *** p<0.01. Standard errors are in parentheses.

Table B: OLS Model: Effect of treatment on economic efficiency controlling for number of group bargaining offers

3.2 Distribution of Gains

To analyze the distribution of lottery tickets for a subject we estimate a similar equation to the efficiency gains analysis, but our left hand side variable is the side payment of lottery tickets that a subject gives/receives after bargaining, as noted in equation 7. The side payments map into probabilities of winning the prize for each subject and have the same interpretation for all subjects. Experimental role is important because the role assigned to a

subject endowed them with different lottery tickets and bargaining power, the dam owner having the most bargaining power.

$$SidePayments_i = \beta_0 + \sum_{j=1}^{J} \beta_j X_{j,i} + \epsilon_i \tag{7}$$

We see from Table C that loss frame statements increase the received side payments for subjects. With additional controls, such as the role subjects play the effect size is less significant. This is because the role effects how language is used in conjunction with bargaining power. In Table D we observe how the analysis done by role changes the interpretation. Dam owners are the ones that largely benefit from the pro-self and loss frame statements though the estimates are rather imprecise. Use of loss frames for fish and wildlife subjects, who do not have bargaining power, increased the amount of side payments distributed by these subjects, though it is noteworthy that few fish and wildlife subjects actually take loss frames (5 out of 54). We also find in Table C that consensus statements are slightly positive, meaning consensus statements lead to greater giving of side payments. We hypothesize that subjects are attempting to close negotiations and making concessions, which are driven by consensus statements but rather precede consensus statements. The pro-self and to a lesser extent the loss frame allows subjects to capture larger side payments though the estimates are rather imprecise, meaning the statistical significance is weak. These effects are consistent with distributive or claiming strategies which use the self or losses as a reference point in negotiating a bigger lottery ball transfer. We control for the number of bargaining offers in Tables E and F. We find the results presented in Tables C and D are robust to including this control.

	(1)	(2)	(3)
Consensus	1.687	1.615	2.080*
	(1.136)	(1.036)	(1.106)
Use of visuals	0.156	-0.262	-0.0847
	(0.722)	(0.682)	(0.894)
Interests	-0.130	0.146	-0.106
	(0.419)	(0.402)	(0.497)
Positions	-0.140	-0.0653	-0.0995
	(0.385)	(0.412)	(0.481)
Pro-self	-2.382	-1.588	-1.592
	(1.560)	(1.344)	(1.445)
Pro-social	0.0987	-0.0871	-0.164
	(0.787)	(0.802)	(0.775)
Gain frame	-0.609	-0.782	-0.657
	(0.914)	(0.957)	(0.942)
Loss frame	-3.228*	-2.296	-2.513
	(1.676)	(1.737)	(1.717)
Role FE	N	Y	Y
Demographic controls	N	Y	Y
Preference controls	N	N	Y
N	162	162	154
R^2	0.0876	0.1993	0.2730

* p<0.1, ** p<0.05, *** p<0.01. Clustered standard errors by group are in parentheses.

Table C: OLS Model: Effect of language on side payments

	Dam Owner	Fish and Wildlife	Local Resident
Consensus	2.872	0.710	0.334
	(2.174)	(2.448)	(1.552)
Use of visuals	-2.708	1.908	0.690
	(2.323)	(1.760)	(1.467)
Interests	-0.147	0.236	1.051
	(0.978)	(1.398)	(0.678)
Positions	-0.121	-1.016	-0.799
	(0.917)	(1.053)	(1.049)
Pro-self	-3.259*	0.433	0.485
	(1.834)	(3.864)	(2.233)
Pro-social	0.195	-1.738	-0.359
	(2.649)	(2.295)	(1.269)
Gain frame	1.269	2.172	-2.535
	(2.268)	(2.752)	(1.743)
Loss frame	-1.437	7.015	-0.148
	(1.678)	(4.589)	(3.005)
Demographic controls	Y	Y	Y
Treatment FE	Y	Y	Y
N	54	54	54
R^2	0.4103	0.2452	0.1800

* p<0.1, ** p<0.05, *** p<0.01.

Table D: OLS Model: Effect of language on side payments by Role

	(1)	(2)	(3)
Number of bargaining offers	0.136	0.0813	0.0866
	(0.122)	(0.0985)	(0.108)
Consensus	1.722	1.633	2.105*
	(1.159)	(1.056)	(1.133)
Use of visuals	0.144	-0.265	-0.0933
	(0.714)	(0.679)	(0.891)
Interests	-0.144	0.137	-0.125
	(0.422)	(0.403)	(0.502)
Positions	-0.144	-0.0696	-0.0931
	(0.386)	(0.411)	(0.488)
Pro-self	-2.428	-1.608	-1.635
	(1.589)	(1.366)	(1.473)
Pro-social	0.109	-0.0832	-0.153
	(0.788)	(0.802)	(0.780)
Gain frame	-0.492	-0.715	-0.579
	(0.938)	(0.969)	(0.953)
Loss frame	-3.283*	-2.334	-2.540
	(1.714)	(1.783)	(1.738)
Role FE	N	Y	Y
Demographic controls	Y	Y	Y
Treatment FE	Y	Y	Y
N	162	162	154
R^2	0.0360	0.124	0.158

* p<0.1, ** p<0.05, *** p<0.01.

Table E: OLS Model: Effect of language on side payments controlling for the number of bargaining offers.

	Dam Owner	Fish and Wildlife	Local Resident
Number of bargaining offers	-0.560	0.480	-0.0867
	(0.391)	(0.434)	(0.310)
Consensus	2.063	0.420	0.285
	(2.219)	(2.455)	(1.580)
Use of Visuals	-3.318	1.890	0.763
	(2.332)	(1.755)	(1.507)
Interests	0.0709	0.395	1.092
	(0.977)	(1.401)	(0.701)
Positions	-0.185	-0.980	-0.796
	(0.906)	(1.051)	(1.062)
Pro-self	-3.119*	1.279	0.591
	(1.813)	(3.929)	(2.291)
Pro-social	-0.117	-1.539	-0.337
	(2.624)	(2.295)	(1.286)
Gain frame	0.518	2.054	-2.611
	(2.299)	(2.746)	(1.784)
Loss frame	-1.045	6.711	-0.252
	(1.679)	(4.585)	(3.063)
Demographic controls	Y	Y	Y
Treatment FE	Y	Y	Y
N	54	54	54
R^2	0.239	0.00550	-0.112

* p<0.1, ** p<0.05, *** p<0.01.

Table F: OLS Model: Effect of language on Side Payments controlling for the number of bargaining offers by Role

3.2.1 Falsification tests

We conduct falsification tests for our significant treatment effects on side payment distribution by role displayed in Figure 2a in the main text. For this test, we use the methods put forth by Young [2018]. The basic idea

	(1)<br>Minimum p-value	(2)<br>Maximum p-value	(3)<br>Randomized p -value
Dam Owner			
Treatment	.06494	.06693	.06677
Fish and Wildlife			
Treatment	.04995	.05095	.05087
Resident			
Treatment	.63736	.64136	.64104
N	27	27	27

"randomization-t" p-values estimated using method of Young (2018) with 1,000 iterations.

Table G: Falsification test summary

of this randomization test is that one randomly reassigns treatment to each observation, after which statistical tests are redone and the new t-statistics are saved. The percentage of the new t-statistics that are above the "true" t-statistic is the p-value of the statistical test. We find support for our conclusions at the approximately same size of p-value provide by a t-test in the main text.

3.3 Crowding Out Communication

In our experiment there is a 20 minute time limit for “cheap talk” and different communication concepts may not work well together for this reason or for reasons of dissonance in the frames adopted by subjects. Here we show descriptive statistics of the cross-section of the significant communication concepts that drive our results to demonstrate that it is different subjects that are using various communication strategies that lead to the outcomes previously described. We show the overlap in gain and loss frames as well as pro-social and pro-self statements.

Frequency of loss statements	Frequency of gain statements 0	1	2	4
0	116	15	2	3
1	12	7	0	0
2	2	2	0	0
3	1	0	0	0
4	2	0	0	0

Table H: Frequency of Loss and Gain Frames

Frequency of pro-social statements	Frequency of pro-self statements 0	1	2	3
0	79	11	5	1
1	37	8	2	1
2	10	2	1	1
3	2	0	0	0
4	1	0	0	0
5	1	0	0	0

Table I: Frequency of Pro-social and Pro-self Statements

Both tables show the majority of subjects are employed in either the gain or loss frames, and similarly in pro-self or pro-social statements.

3.4 Content and Economic Efficiency

To further analyze the content of the communication sessions we code segments of dialogue using the code book provided in Section 4. The results in Table J measure the effect of the frequency of the type of coded communication on economic efficiency. We find robust results for the positive effect of *use of visuals* and *gain frame* on value creation as measured by economic efficiency. An increase in the frequency of either of these concepts leads to a gains between 6-13% of economic efficiency. This is both statistically and economically significant to bargaining outcomes. We find a weaker effect on *consensus* statements though the positive sign on the coefficient is expected.

	(1)	(2)	(3)
Consensus	0.0680*	0.0387*	0.0388
	(0.0377)	(0.0226)	(0.0268)
Use of visuals	0.123***	0.0963***	0.0770***
	(0.0308)	(0.0238)	(0.0229)
Interests	-0.0158	0.00462	0.00718
	(0.0216)	(0.0181)	(0.0182)
Positions	-0.0249	-0.0195	-0.0106
	(0.0198)	(0.0167)	(0.0173)
Pro-self	0.0272	0.0147	0.00296
	(0.0428)	(0.0396)	(0.0556)
Pro-social	-0.00468	0.00354	0.00991
	(0.0262)	(0.0205)	(0.0201)
Gain frame	0.133***	0.0849***	0.0661**
	(0.0350)	(0.0259)	(0.0264)
Loss frame	0.0397	0.0310	0.0342
	(0.0341)	(0.0245)	(0.0302)
Session FE	N	Y	Y
Demographic and preference controls	N	N	Y
N	162	162	154
R^2	0.1922	0.5115	0.5372

* p<0.1, ** p<0.05, *** p<0.01. Clustered standard errors by group are in parentheses.

Table J: OLS Model: Effect of language on economic efficiency

Interestingly, there is a clear divergence of the language used that creates value for the group versus language used to create value for the individual. In our setup, these need not be different since the decision of the dam site decision and transfers can be made to capture greater individual gains as well as group gains. Some of these effects are driven by priming while others are endogenous to the subjects and group dynamics.

4 Codebook for Content Analysis

Dialogue from the communication sessions are coded to categories provided in the codebook in Table K. Some of the concepts that are coded from the experiment, such as *dissensus*, are not used in the analysis because they occur very infrequently and are co-linear with other controls. Identification of effects for these variables is confounded with the other controls because they occur so infrequently.

Code	Description
Agreement Moments	
Consensus	Subject speaks in a manner that indicates consensus is being reached.
Dissensus	Subject speaks in a manner indicate there is not agreement among the members.
Argument Framing	
Gain frame	Subjects refer to outcomes or choices which benefit themselves or the group. Gains may refer to lottery tickets or other intangible gains.
Loss frame	Subjects refer to outcomes or choices which are detrimental to themselves or the group. Losses may refer to lottery tickets or other intangible damage.
Agree to disagree	Recognition that some parties will not find common ground.
Use of Data or Visuals	
Use of visuals	Subjects specifically mention aesthetics or reference visual aids.
Negotiation Strategies	
Interests	Subjects describes an interest during the negotiation.
Positions	Subjects describes a position during the negotiation.
Social Preferences	
Pro-self (Exclusive)	Subjects frame arguments in a pro-self manner; referring to themselves or outcomes that impact them.
Pro-group (Inclusive)	Subjects frame arguments in a social context- includes the group in reference to the outcomes or choices.
Power Frame	
Authority	Subject talks about power of roles i.e. Dam owner talking about final authority; other players recognizing final authority in negotiation.

Table K: Codebook

5 Experiment Instructions

Instructions

In this experiment you and two other players will be negotiating to make a group decision.

You will each be given a card that describes the role you play for this experiment. You may not share the physical card with other members of your group. It is up to you to decide how much information you would like to share from your card with the other group members.

Each participant is assigned an initial amount of points, depending on what decision is made by the group. These are listed in the table on your Role Description card. These points represent your chance of winning the final $20 cash prize at the end of the experiment. If your card says "60" for an option, then you have a 60% chance to win the final prize if the group picks that option (without transfers). You may offer to divide a portion of your points between one or both of the other players as part of your negotiation offers.

One of the players will be the "controller" of the game and may end the negotiation at any time and pick whichever option they think is best. We'll talk more about this before the game begins.

The Game

There are two stages in this game.

Stage 1: In the first stage, you will talk with the other group members for up to 20 minutes. This will be recorded, so please say your role name before making any comments. This will help the researchers later.

Stage 2: In the second stage, participants engage in a 10-minute negotiation period. During this stage, you may only exchange written offers and counteroffers to reach an agreement. You will use the provided "Offer Sheet" to record offers and submit to the monitor.

You and the other players may agree to transfer points to other players in order to select one option. Again, these points represent your chances of winning the final $20 cash prize.

Important Things to remember:

- Your number of points at the end of the experiment represent the chance you have to win the final $20 prize.

- Points are awarded based on the option picked by the group.
- Points may be traded to come to an agreement with other players.
- All transfers must add up to zero for an offer to be valid.
- All three group members must agree on an option, unless the "controller" ends negotiations.
- If the controller ends negotiations, they may choose whichever option they would like. Points awarded are the default value on your card. There are no point transfers.
- There will be no physical threats or other inappropriate behavior during the session

Example: Making an offer

In this example, let's assume we know everyone's payoffs for every option. You will only know your own payoffs during the game, unless the other players tell you their payoffs. Player A proposes an option that we call "Option 1" here. Based on the role-specific points and no point transfers, Player A would get 30 points, Player B would get 20 points, and Player C would get 15 points.

Player A thinks 30 points is not enough compensation to accept this option outright, but would accept this option if they received a total payoff of 55 points.

So, Player A asks Player B to pay them 15 points and Player C to pay them 10 points to accept this option. This would give Player A the payout of 55 total points they want for this option.

Here's what the offer sheet would look like for this offer:

	Point Transfer (these should add to zero)		
Option:	Player A	Player B	Player C
Option 1	+ 25	-15	-10

If the group accepts this option (all group members must agree, or no transfer occurs), Player A would receive 55 points (30 initial points + 25 transfer points), Player B would receive 5 points (20 initial points – 15 transfer points), and Player C would receive 5 points (15 initial points – 10 transfer points). Each player would now have the following chance to win the final prize:

Player A: 55 points = 55% chance to win
Player B: 5 points = 5% chance to win
Player C: 5 points = 5% chance to win

6 Scenario Description

The "Litchberg" dam was built in 1890 to provide power to a small textile manufacturing mill. The mill closed 40 years ago and the mill buildings were abandoned. Although it is no longer being used to power the mill, the 10' tall dam is still in place. The dam is in a central location in the small rural township of Litchberg. There is a 5-acre pond created by the dam that is used by the community for fishing. In addition, there are properties around the pond with waterfront property. Some within the community have been researching whether the dam could provide hydropower for the town. The river was an important river for migratory fish such as Alewife, Shad, Herring, and Atlantic Salmon. These fish species spend the majority of their life in salt water but migrate up the rivers to lay their eggs and reproduce. The dam is the first dam on the river and blocks all fish passage to the wetlands upstream that would be ideal breeding grounds for the fish. Fish populations in the area have declined significantly and the local fishing industry which used to be an important part of the economy has declined.

The dam was recently identified as a high hazard dam because of the age and the potential impact on downstream homes if the dam would break. The dam owner has been given 1 year to respond to the deficiency notice.

7 Dam Site Options

- Repair Dam
  The dam remains in place and repairs are made that are consistent with historic character of dam. Because of the 10'dam, fish cannot

pass upstream to reproduce. The impoundment water elevation stays the same, and the potential for hydropower still exists. This is the least cost option.

- Repair Dam + Denil Fish Ladder
  The dam remains in place, repairs are made to the dam and a new Denil Fish Ladder is constructed alongside the dam. A Denil fish ladder is a rectangular concrete chute that is made of a sequence of baffles and resting pools. A newly constructed denil fish ladder can provide up to 30% fish passage. Because the dam remains in place, the impoundment water elevation stays the same and the potential for hydropower still exists.

- Nature like fish ladder
  The dam elevation is lowered 3' and soil and rocks are terraced from the downstream side of the dam until the elevation reaches the same height of the dam. Because of the fill material, the dam is no longer visible. Nature like fish ladders can provide up to 60% fish passage. Because the dam is lowered, the impoundment water elevation is also lowered 3' resulting in a reduced hydropower potential.

- By – Pass channel
  The dam remains in place, repairs are made to the existing structure and a bypass channel is dug around the dam. The bypass channel can provide up to 60% fish passage. The impoundment elevation will stay the same and the potential for hydropower will still exist.

- Full removal
  The dam will be entirely removed including the abutments to allow for a free-flowing river. This alternative would provide 100% fish passage. The impoundment elevation would drop down by 10' to the width of the river channel downstream. There would be no hydropower potential in the future.

The cost for each of these options is increasing, the least cost option is to repair the dam, then adding the fish ladder, then the by-pass channel, and the highest cost option is to remove the dam. The residents and owner of the dam do not fully incur the costs because of federal support to build fish ladders and remove dams.

8 Experiment Visuals

Below are the visuals provided to the experimental subjects during the bargaining game. They were provided individually on poster board that was approximately four feet long by three feet wide.

Repair Dam

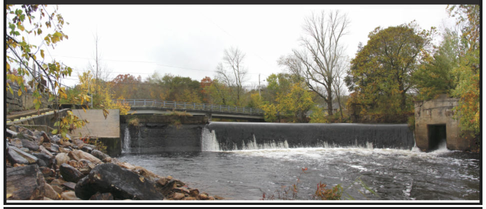

New Denil Fish Ladder

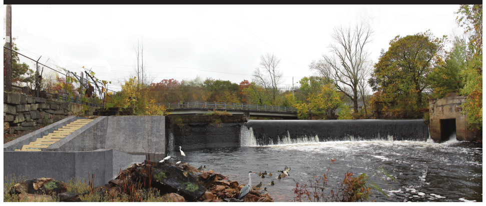

Nature Like Fish Ladder

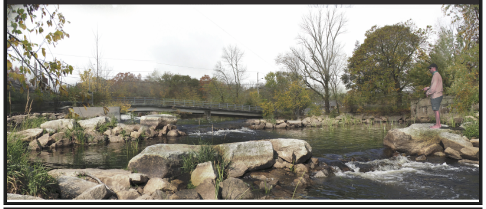

By-Pass Channel

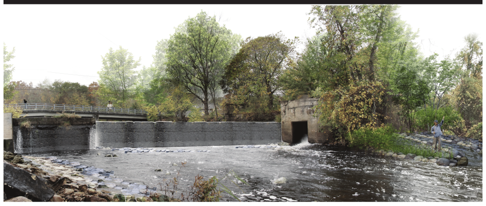

Full Dam Removal

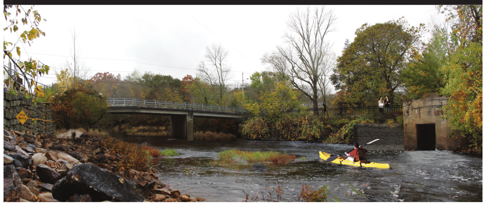

9 Roles

The roles and payoff information below was private to the subject assigned to that role.

Dam Owner - Controller:

You are a resident that lives upstream from the dam on the impoundment. The dam and remaining mill buildings are on your property, giving you final say on the decision of the Litchberg Dam's fate. People love to fish at the impoundment and you taught your kids to swim in the impoundment.

You are an avid fisherman and like to fish in both fresh and salt water. You are aware of the decline in the fish populations in the ocean, but you aren't clear about what has caused the decline. You would like the dam to remain. You enjoy spending time with your family at the impoundment and would like future generations to do the same. You'd like to maintain the option for future hydropower since it might help offset costs associated with maintenance of the dam, but also are aware the initial cost of building a hydropower plant is high.

Based on the costs and preferences for each of these options you have payout schedule.

Payoffs:

Option:	Payoff
Repair	65
Repair + Denil Fish Ladder	55
Nature-Like fish ladder	35
By-pass channel	40
Dam Removal	0

Resident:

You are a resident that lives downstream of the dam. In addition to enjoying having water-front property, you believe that the dam and impoundment are an important part of the communities' local identity. You enjoy the sight of the dam, but you are concerned about the potential breach of the dam and the impact it would have on your property. You like to take your boat out on the river. You sometimes drive it upstream and launch at the impoundment on weekends, where you often bump into old friends from high school. You wonder what it would be like to drive your boat upstream if the dam did not block the river, making it less of hassle hauling your boat in and out of the river. If a By-pass channel is built you can drive your boat upstream, and there is still an option for hydropower in the future while also providing greater fish passage. You would like to maintain some potential for hydropower because it is a renewable source of energy and a report at the town office said it could reduce electricity rates.

Based on the costs and preferences for each of these options you have payout

schedule.

Payoffs:

Option:	Payoff
Repair	5
Repair + Denil Fish Ladder	5
Nature-Like fish ladder	25
By-pass channel	25
Dam Removal	10

Fish and Wildlife:

You are a representative from Fish and Wildlife. You serve on the local Conservation Commission and believe preserving and restoring nature is important for communities. Fish passage needs to be restored. Improved fish passage is necessary for proper stock rebuilding efforts to be effective. The dam is no longer serving any functional purpose for the town, while continuing to harm the local environment and wildlife. You have heard rumors about potential hydropower production on the dam, but doubt there will be any significant power that could be generated from such a small dam. A Nature Like Fish ladder would improve fish passage, but would reduce potential for hydropower due to a 3' drop in impoundment water levels. This may also reduce the impact of floods on downstream property owners.

Based on the costs and preferences for each of these options you have payout schedule.

Payoffs:

Option:	Payoff
Repair	0
Repair + Denil Fish Ladder	15
Nature-Like fish ladder	40
By-pass channel	20
Dam Removal	70

References

Evert Van der Vliert. *Complex interpersonal conflict behaviour: Theoretical frontiers*. Psychology Press, 2013.

Riley E Dunlap, Kent D Van Liere, Angela G Mertig, and Robert Emmet Jones. New trends in measuring environmental attitudes: measuring endorsement of the new ecological paradigm: a revised nep scale. *Journal of social issues*, 56(3):425–442, 2000.

Alwyn Young. Channeling fisher: Randomization tests and the statistical insignificance of seemingly significant experimental results∗. *The Quarterly Journal of Economics*, 134 (2) : 557 − −598, $November$2018. doi : 10.1093/qje/qjy029.URLhttps://doi.org/10.1093/qje/qjy029.
